# Control of Allergic Rhinitis and Asthma Test for Children (CARATkids): A systematic review and meta‐analysis of its measurement properties

**DOI:** 10.1111/pai.70191

**Published:** 2025-09-02

**Authors:** Hadla Sami El Didi, Ana Margarida Pereira, Cristina Jácome, Rita Amaral, Gustavo F. Wandalsen, Joyce Emons, Stefania La Grutta, Giovanna Cilluffo, Sehra Birgül Batmaz, Daniela Linhares, Dirceu Sole, Bernardo Sousa‐Pinto, João Almeida Fonseca, Rafael José Vieira

**Affiliations:** ^1^ Department of Community Medicine, Information and Health Decision Sciences, Faculty of Medicine, RISE‐Health University of Porto Porto Portugal; ^2^ School of Health Polytechnic of Porto Porto Portugal; ^3^ Escola Paulista de Medicina Universidade Federal de São Paulo São Paulo Brazil; ^4^ Division of Pediatric Respiratory Medicine and Allergology, Department of Pediatrics Erasmus MC University Medical Center Rotterdam The Netherlands; ^5^ Institute of Translational Pharmacology National Research Council Palermo Italy; ^6^ Department of Earth and Marine Sciences, DiSTeM University of Palermo Palermo Italy; ^7^ CoNISMa National Inter‐University Consortium for Marine Sciences Rome Italy; ^8^ Department of Pediatric Allergy and Clinical Immunology Tokat State Hospital Tokat Turkey

**Keywords:** Allergic rhinitis, asthma, CARATkids, COSMIN, patient‐reported outcomes, psychometrics

## Abstract

Control of Allergic Rhinitis and Asthma Test for Children (CARATkids) is the first patient‐reported outcome measure (PROM) designed to assess both allergic rhinitis and asthma simultaneously in children aged 6 to 12 years. CARATkids has been validated in several languages and countries, highlighting the need for a review of its psychometric properties. This study aims to evaluate the measurement properties of CARATkids. This systematic review follows PRISMA and COSMIN guidelines. A systematic search was performed across three databases (Ovid/MEDLINE, Web of Science, and Scopus in October 2023, updated in June 2025). We included studies focused on the development, cultural adaptation, or validation of CARATkids, as well as studies comparing CARATkids with other PROMs. We evaluated the quality of CARATkids development, the methodological quality of primary studies, the overall rating, and the certainty of evidence for each CARATkids measurement property and performed a meta‐analysis of its measurement properties. Our search retrieved 193 results. We included nine studies. CARATkids displayed sufficient content validity. Regarding internal consistency, we found a meta‐analytical Cronbach alpha of 0.81 (95% CI = 0.79; 0.83). CARATkids displayed sufficient reliability (meta‐analytical intraclass correlation coefficient 0.86 [95% CI = 0.61; 0.96]). The minimal clinically important difference was 2.76. Construct validity had sufficient evidence for most correlations, with absolute meta‐analytical Spearman coefficients from 0.37 to 0.71. Responsiveness showed strong correlations between CARATkids and most outcome measurement instruments. These findings support CARATkids as a suitable tool for assessing asthma and allergic rhinitis in children aged 6 to 12 years who present both conditions simultaneously.

**Key message**

CARATkids is a reliable and valid patient‐reported outcome measure for assessing asthma and allergic rhinitis control in children, supporting its clinical use for monitoring and guiding treatment decisions.

## 1 INTRODUCTION

Patient‐reported outcome measures (PROMs) quantify the impact of health conditions and treatments from the patients' perspective.^1^ They provide insights into several aspects of disease, including symptom burden, well‐being, and quality of life,^2^ informing clinical decisions and resource planning.^3^

In pediatric care, the importance of understanding patients' perceptions of their diseases and healthcare experience is increasingly recognized.^4^ PROMs empower children to actively participate in managing their conditions.^5^ This is especially relevant in asthma and allergic rhinitis, as these chronic conditions present with variable symptoms and triggers, so that PROMs may help tailor treatment and improve long‐term management.

There are several PROMs available to assess the control of asthma or allergic rhinitis in children, such as the Childhood Asthma Control Test (cACT),^6^ the Pediatric Quality of Life Asthma Questionnaire,^7^, ^8^ the Asthma Therapy Assessment Questionnaire for Children (ATAQ‐Children),^9^ the Visual Analogue Scale (VAS) for asthma and allergic rhinitis,^10^ the Total Nasal Symptom Score (TNSS),^10^ and the Control of Allergic Rhinitis and Asthma Test for Children (CARATkids).^11^ However, only the latter assesses both conditions simultaneously. The Allergic Rhinitis and Its Impact on Asthma (ARIA) group recommends that both conditions be assessed using a single tool, since allergic rhinitis is frequently associated with asthma, representing both a risk factor and a contributor to poor asthma control.^12^ To the best of our knowledge, CARATkids is the only PROM assessing the control of both asthma and allergic rhinitis in children. Developed from the Control of Allergic Rhinitis and Asthma Test (CARAT) questionnaire for adults,^13^ the pediatric version consists of 13 questions (8 for children and 5 for caregivers).^11^, ^14^ Its score ranges from 0 (best control) to 13 (worst control) points, with scores of 3 or lower indicating good disease control, and scores of 6 or higher identifying uncontrolled disease.^15^

Besides Portugal,^11^, ^14^ where this tool was created, CARATkids has already been applied and validated in several languages and countries, such as Brazil,^15^ Turkey,^10^ Italy,^16^, ^17^ and the Netherlands.^18^ Its widespread use prompts the need for a systematic assessment of its measurement (psychometric) properties. Therefore, the purpose of this systematic review was to objectively evaluate the measurement properties of CARATkids using the COnsensus‐based Standards for the selection of health status Measurement Instruments (COSMIN) guidelines for systematic reviews of PROMs.^19^, ^20^, ^21^

## 2 METHODS

### 2.1 Study design

This systematic review was reported according to the recommendations of the Preferred Reporting Items for Systematic Reviews and Meta‐Analyses (PRISMA)^22^ and the recommendations of the COSMIN methodology for systematic reviews of PROMs.^19^, ^20^, ^21^

### 2.2 Selection criteria

We included original studies that (i) involved children aged 6 to 12 years with asthma and/or allergic rhinitis, corresponding to the target population for the application of the PROM, and (ii) focused on its development, cultural adaptation, or validation, or which employed the CARATkids alongside another PROM to assess allergic rhinitis and/or asthma control. Conference abstracts were excluded per COSMIN recommendations. No limitations were applied concerning the publication date or language.

### 2.3 Search strategy

We searched Ovid/MEDLINE, Web of Science, and Scopus in October 2023, with an update in June 2025. The search strategy was created by search experts from the Porto Associate Center of Cochrane Portugal and the Portuguese GRADE network (Table S1). We also screened reference lists and manually searched Google Scholar for additional relevant studies (including relevant gray literature).

### 2.4 Study selection

After removing duplicates, two authors independently evaluated articles' titles and abstracts. Reports that were not excluded were then independently assessed in their full content. When access to any of the publications was not possible by other means, efforts were made to contact the original investigators. Lack of access to publications did not lead to the exclusion of any studies. Conflicts between the authors were resolved by consensus.

### 2.5 Data extraction

Two authors independently extracted data from each included primary study using a custom‐built form (Data S2), including sample size, age and gender distribution of participants, prevalence of patients with allergic rhinitis and/or asthma, country and language of questionnaire administration. Additionally, we extracted data on the measurement properties reported for CARATkids in each primary study.

### 2.6 Quality assessment

Quality assessment followed the COSMIN methodology for assessing the content validity of PROMs—User Manual^23^ and the COSMIN guideline for systematic reviews of PROM.^19^ Two authors independently assessed (i) the quality of the PROM development, (ii) the methodological quality of primary studies, (iii) the overall rating, and (iv) the certainty of evidence for each measurement property (Figure S1).

The development of CARATkids was assessed based on the PROM design and cognitive pilot testing, as described in the COSMIN methodology for assessing the content validity of PROMs—User Manual.^23^ Each item was classified into “Very Good,” “Adequate,” “Doubtful,” or “Inadequate.” For comparison, the same assessment was performed for three other pediatric asthma PROMs, namely cACT, CAQ‐B, and TRACK.

The methodological quality of primary studies (including those concerning PROM development) was assessed using the COSMIN risk of bias checklist,^19^ with ratings from “Very Good” to “Inadequate.” For each measurement property, the rating was based on the lowest graded item within each measurement property.

The overall rating concerns the quantitative results of each measurement property compared with pre‐established criteria for good measurement properties (typically predefined cut‐offs).^19^ The results for each measurement property were rated as “Sufficient (+),” “Insufficient (−),” or “Indeterminate (?),” based on their alignment with these criteria. Assessed measurement properties included content validity, internal consistency, reliability, construct validity, and responsiveness. None of the included studies assessed the structural validity of CARATkids. Content validity was assessed using five relevance criteria, one for comprehensiveness, and two for comprehensibility.^23^ The remaining properties were assessed through comparison with pre‐established criteria for good measurement properties (typically predefined cut‐offs),^19^ available in Table S2. The results for each measurement property were rated as “Sufficient (+),” “Insufficient (−),” or “Indeterminate (?),” based on their alignment with these criteria.

Finally, the certainty of evidence was rated as high, moderate, low, or very low based on the Grading of Recommendations Assessment, Development and Evaluation (GRADE) methodology, which considers the methodological quality of the studies, inconsistency, imprecision, and indirectness.^19^, ^22^, ^23^, ^24^, ^25^

### 2.7 Data analysis

We conducted meta‐analyses of Cronbach's alpha (internal consistency), intraclass correlation coefficients (reliability), and Spearman correlation coefficients (construct validity and responsiveness). Other properties, such as measurement error, were not analyzed due to insufficient data.

We applied a random‐effects model, using the restricted maximum likelihood method. Whenever studies did not report precision measures, variances were estimated following methods reported elsewhere.^13^ Therefore, for performing meta‐analysis of Spearman correlation coefficients and ICCs, coefficients were firstly transformed according to the formula $0.5\times \ln\;\left(\frac{1+\text{correlation}\;\text{coefficient}}{1-\text{correlation}\;\text{coefficient}}\right)$, with their variances being estimated by $\frac{1}{n-3}$ ^26^; meta‐analytical results were then back transformed to the natural scale. For Cronbach alphas, variances were estimated based on computed confidence interval limits.^27^

Heterogeneity was assessed using the *I* ^2^ statistic and the *p*‐value from the Q‐Cochran statistic, with *I* ^2^ > 50% and *p* < .10 deemed to represent substantial and significant heterogeneity. In the presence of substantial heterogeneity, leave‐one‐out sensitivity analyses were performed.

All analyses were performed using R (version 4.4.1; R Core Team 2024).

## 3 RESULTS

### 3.1 Studies selection

**FIGURE 1:**
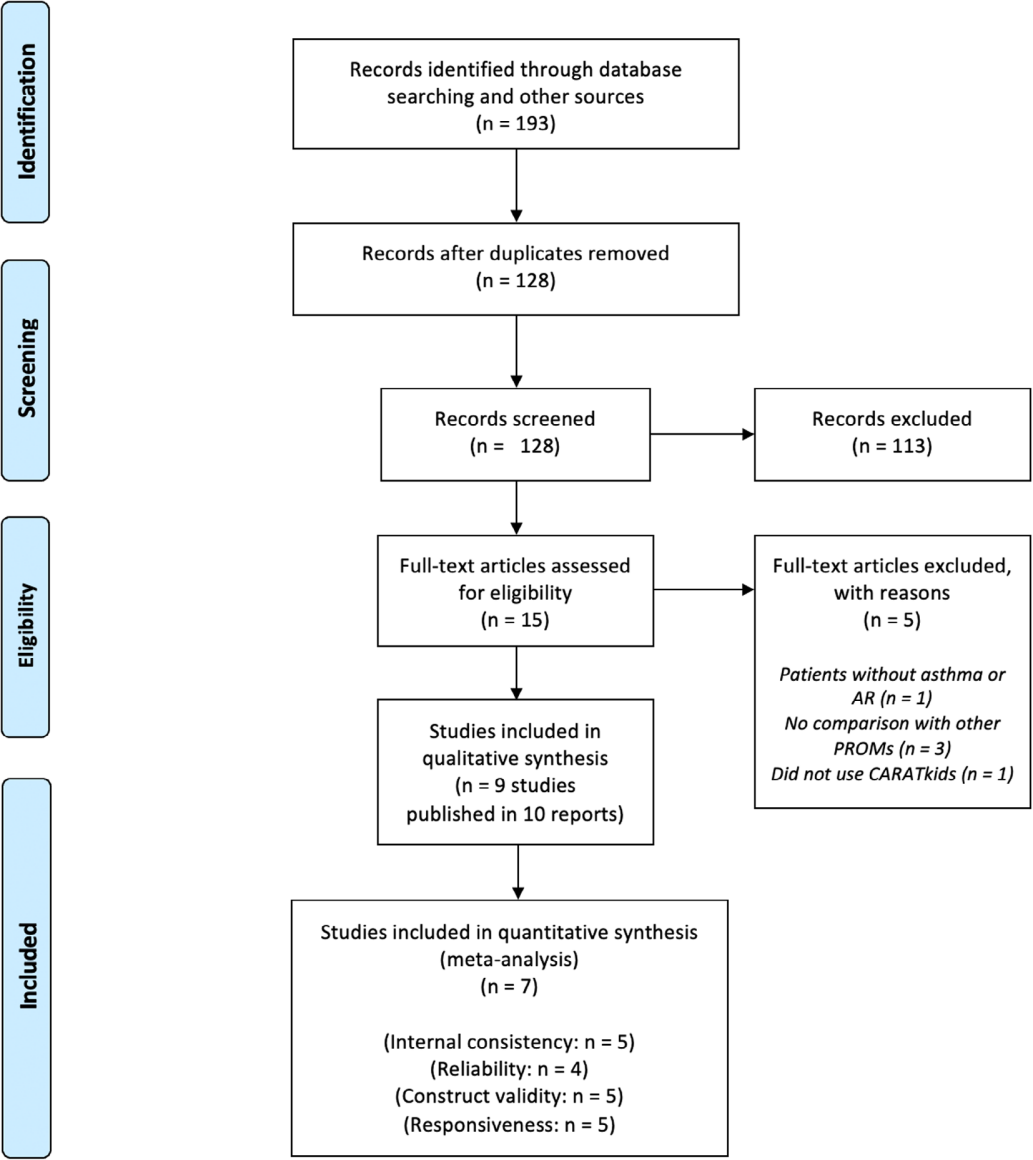
Preferred Reporting Items for Systematic Reviews and Meta‐Analyses (PRISMA) flow diagram illustrating the studies' selection process. Records refer to titles and abstracts identified through database or other searches; reports are full‐text articles or documents retrieved for detailed evaluation; studies are unique investigations (e.g., experimental or observational studies) that may be published/described in one or more reports.

Our search yielded a total of 193 results (Figure 1). After removing duplicate entries, 128 unique studies remained for initial screening. Of those, 15 studies were evaluated based on their full texts. In total, nine original studies (10 reports, since the development study was published in two reports^11^, ^28^) were included in the systematic review (Table 1).^10^, ^11^, ^14^, ^15^, ^16^, ^17^, ^18^, ^29^, ^30^

**TABLE 1:** Characteristics of the included studies (*n* = 771 participants).

	*N*	Age mean (SD)	Females *N* (%)	Country	Language
Borrego 2013^a^	29	8	11 (37.9)	Portugal	European Portuguese
Linhares 2014	113^b^	8.75 (1.86)	44 (38.9)	Portugal	European Portuguese
Resende 2015	47	9.34 (2.34)	13 (27.7)	Portugal	European Portuguese
Emons 2017	55	8.6 (1.69)	29 (52.7)	Netherlands	Dutch
Amaral 2017	102	9.1 (1.8)	55 (53.9)	Brazil	Brazilian Portuguese
Batmaz 2018	174	8.72 (2.15)	78 (44.8)	Turkey	Turkish
Cilluffo 2019	112	8.3 (1.63)	37 (33.0)	Italy	Italian
Tosca 2020	88	9.9	30 (34.1)	Italy	Italian
Mata 2025	51	9	22 (43.1)	Portugal	European Portuguese

Abbreviation: SD, standard deviation.

^a^ Original CARATkids development study.

^b^ 101 patients filled out the CARATkids questionnaire in both visits.

### 3.2 Characteristics of included studies

Table 1 provides a summary of the included studies. Five studies (published in six reports) employed the Portuguese version of CARATkids,^11^, ^14^, ^15^, ^28^, ^29^, ^30^ including one adapted to Brazilian Portuguese.^15^ Two studies used the Italian version,^16^, ^17^ one study applied the Dutch version^18^ and another used the Turkish version.^10^ One study compared an electronic Portuguese version of CARATkids against the paper version.^30^ In total, 771 children were assessed, with a mean age ranging from 8 to 9.9 years old. One Italian study^17^ and the Dutch study^18^ also included adolescents, but only the Italian study reported results for each subgroup. Aside from the Dutch and one of the Portuguese studies,^29^ participants in all studies presented both AR and asthma.

### 3.3 Methodological quality of primary studies

The methodological quality ratings for each psychometric property varied within each study. Most of the properties were rated as either “Very good” or “Adequate,” suggesting a low overall risk of bias (Table 2).

**TABLE 2:** Methodological quality of studies on the measurement properties of the Control of Allergic Rhinitis and Asthma Test for Children (CARATkids).

	Structural validity	Internal consistency	Reliability	Measurement error	Construct validity	Responsiveness
Linhares 2014	—	V	V	—	A	A
Resende 2015	—	—	—	—	A	—
Emons 2016	—	V	V	V	A	A
Amaral 2017	—	V	V	V	A	A
Batmaz 2018	—	V	V	V	A	A
Cillufo 2019	—	—	—	—	A	A
Tosca 2020	—	—	—	—	A	—
Mata 2025	—	V	—	—	—	—
Overall risk of bias	Low	Low	Low	Low	Low	Low

*Note*: A = adequate; V = very good.

The quality of PROM development is presented in Table S3. This assessment was based on the development study (published in two reports^11^, ^28^). Regarding general design requirements, most criteria were rated as “Very good,” except for sample representativeness, which was rated as “Inadequate.” That is because the development was based on consensus meetings with experts, with no explicit involvement of children or their caregivers in this phase. Consequently, the design of CARATkids was rated “Inadequate,” reflecting the lowest assigned rating. For the cognitive interview study, sample representativeness was considered “Very good.” Comprehensibility was deemed “Doubtful,” since meetings were recorded but not transcribed verbatim. As a comparison, we also assessed the quality of the development of cACT, CAQ‐B, and TRACK. The development of these three PROMs was deemed “Inadequate” as well (Table S4).

### 3.4 Measurement properties of CARATkids

**FIGURE 2:**
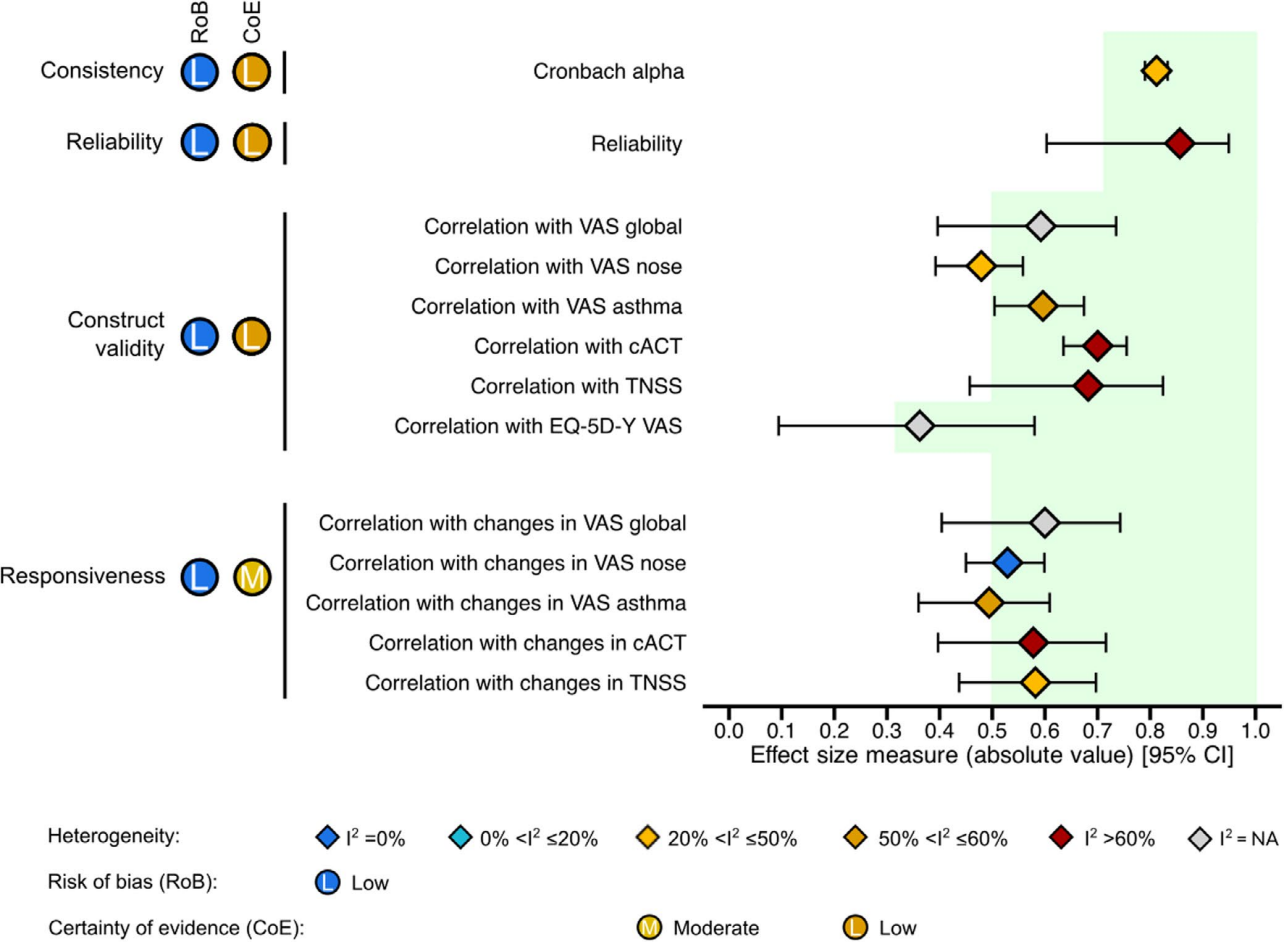
Main meta‐analytical results on the properties of the Control of Allergic Rhinitis and Asthma Test for Children (CARATkids). Light green areas indicate the range of good results according to the COSMIN guidelines, that is, values that meet the criteria for sufficient measurement properties: Cronbach's alpha ≥0.70 for internal consistency, intraclass correlation coefficient (ICC) ≥0.70 for reliability, and Spearman's correlation coefficient ≥0.70 for similar constructs or ≥0.50 for related but dissimilar constructs in the evaluation of construct validity and responsiveness. cACT, Childhood Asthma Control Test; CI, confidence interval; TNSS, Total Nasal Symptom Score; VAS, visual analogue scale.

All measurement properties were assessed at least once, except for structural validity, cross‐cultural validity, and criterion validity. For the latter, comparisons between CARATkids and other PROMs were interpreted as evidence for construct validity (and not criterion validity), in accordance with COSMIN guidelines.^19^ Overall, we found evidence for sufficient content validity, reliability, construct validity, and responsiveness. Internal consistency was rated indeterminate since structural validity was not assessed by any study (Tables S5 and S6). Meta‐analytical details can be seen in Table 3 and are summarized in Figure 2.

**TABLE 3:** Meta‐analytical results for the consistency, reliability, construct validity, and responsiveness of the Control of Allergic Rhinitis and Asthma Test for Children (CARATkids).

	*N* primary studies	*N* participants	Meta‐analytical result (95% CI) [*I* ^2^; *Q*‐Cochran *p*‐value]
Internal consistency—Cronbach alpha	5	495	0.81 (0.79; 0.83) [23.6%; .320]
Reliability—ICC	4	439	0.86 (0.61; 0.96) [97.4%; <.001]
Construct validity			
Correlation with VAS global	1	55	0.60 (0.40; 0.74) [−]
Correlation with VAS nose	4	432	0.49 (0.40; 0.57) [37.7%; .217]
Correlation with VAS asthma	4	432	0.60 (0.51; 0.68) [57.5%; .070]^a^
Correlation with cACT	5	544	−0.71 (−0.76; −0.64) [67.7%; .020]
Correlation with TNSS	2	276	0.69 (0.47; 0.83) [93.0%; <.001]
Correlation with EQ‐5D‐Y VAS	1	47	−0.37 (−0.59; −0.10) [−]
Responsiveness			
Correlation with VAS global	1	55	0.60 (0.40; 0.74) [−]
Correlation with VAS nose	4	432	0.53 (0.45; 0.60) [0%; .73]
Correlation with VAS asthma	4	432	0.49 (0.36; 0.61) [55.9%; .076]^b^
Correlation with cACT	5	544	−0.58 (−0.72; −0.40) [83.5%; <.001]^c^
Correlation with TNSS	2	276	0.58 (0.44; 0.70) [48.6%; .163]

Abbreviations: cACT, childhood asthma control test; CI, confidence interval; ICC, intraclass correlation coefficient; TNSS, Total Nasal Symptom Score; VAS, visual analogue scale.

^a^ In leave‐one‐out sensitivity analysis, excluding Batmaz 2018: 0.56 (95% CI = 0.49; 0.63); *I* ^2^ = 4.9%.

^b^ In leave‐one‐out sensitivity analysis, excluding Amaral 2017: 0.56 (95% CI = 0.47; 0.63); *I* ^2^ = 0%.

^c^ In leave‐one‐out sensitivity analysis, excluding Batmaz 2018: −0.51 (95% CI = −0.59; −0.41); *I* ^2^ = 0%.

#### 3.4.1 Content validity

The assessment of the content validity was based on the development study^28^ and our own assessment (Table S5). We rated content validity as “Sufficient,” but the certainty of evidence supporting this evaluation is very low, due to the absence of independent studies assessing content validity.

#### 3.4.2 Internal consistency

Internal consistency was assessed in five studies,^10^, ^14^, ^15^, ^18^, ^30^ with all studies reporting a Cronbach's alpha >0.70. However, studies did not display at least low evidence for sufficient structural validity. Therefore, the internal consistency was rated as “Indeterminate” (Table S6). Regardless, we quantitatively summarized the results for internal consistency (Table 3). CARATkids displayed a meta‐analytical Cronbach alpha of 0.81 (95% CI = 0.79; 0.83) with low heterogeneity (*I* ^2^ = 23.6%) (Certainty of Evidence: Low).

#### 3.4.3 Reliability

Reliability was evaluated in four studies,^10^, ^14^, ^15^, ^18^ all of which presented an ICC > 0.70. Based on this, this psychometric parameter was considered “Sufficient.” Meta‐analytical results for reliability revealed high reliability, with an ICC = 0.86 (95% CI = 0.61; 0.96) with severe heterogeneity (*I* ^2^ = 97.4%) (Certainty of Evidence: Low) (Table 3, Table S6).

#### 3.4.4 Measurement error

Measurement error was assessed in three studies,^10^, ^15^, ^18^ with a low risk of bias. The reported smallest error of measurement of CARATkids was 1.3,^15^ while the minimal clinically important difference was 2.76.^18^

#### 3.4.5 Construct validity

Construct (convergent) validity was assessed in seven studies,^10^, ^14^, ^15^, ^16^, ^17^, ^18^, ^29^ but one of them did not report correlation coefficients nor areas under the ROC curve.^17^ We found mostly “Sufficient” evidence for construct validity, except for correlations with VAS nose. Certainty of evidence ranged from low to high (Table S6).

In quantitative synthesis, CARATkids displayed strong correlations with most instruments, including VAS global (one study^18^), VAS asthma (four studies^10^, ^14^, ^15^, ^18^), cACT (five studies^10^, ^14^, ^15^, ^16^, ^18^), and TNSS (two studies^10^, ^15^) (Table 3). Neither EQ‐5D‐Y (one study^29^) nor VAS nose (four studies^10^, ^14^, ^15^, ^18^) yielded a strong correlation with CARATkids (Table 3). Absolute meta‐analytical Spearman coefficients for the correlation between CARATkids and each measurement instrument ranged from 0.37 to 0.71 (Table 3). Substantial heterogeneity was observed for VAS asthma (*I* ^2^ = 57.5%, in leave‐one‐out sensitivity analysis after excluding the study Batmaz 2018: *I* ^2^ = 4.9%), cACT (*I* ^2^ = 67.7%), and TNSS (*I* ^2^ = 93.0%).

Four studies reported areas under the ROC curve,^10^, ^14^, ^15^, ^16^ all of them displaying an area under the ROC curve ≥0.70 (Table S7).

#### 3.4.6 Responsiveness

Five studies^10^, ^14^, ^15^, ^16^, ^18^ provided evidence for responsiveness (Table S6). We found “Sufficient” responsiveness with VAS nose (four studies^10^, ^14^, ^15^, ^18^), VAS global (one study^18^) and cACT (five studies^10^, ^14^, ^15^, ^16^, ^18^), and “Inconsistent” responsiveness with VAS asthma (four studies^10^, ^14^, ^15^, ^18^) and the TNSS (two studies^10^, ^15^). Certainty of evidence was moderate to high (Table S6).

In quantitative synthesis, CARATkids displayed high responsiveness when compared to changes in the majority of assessed outcome instruments, except for correlation with VAS asthma: 0.49 (95% CI = 0.36; 0.61). For the other PROMs, meta‐analytical correlation coefficients for responsiveness ranged from 0.53 to 0.60 (Table S6). Substantial heterogeneity was observed for VAS asthma (*I* ^2^ = 55.9%, in leave‐one‐out sensitivity analysis after excluding the study Amaral 2017: *I* ^2^ = 0%), cACT (*I* ^2^ = 83.5%, in leave‐one‐out sensitivity analysis after excluding the study Batmaz 2018: *I* ^2^ = 0%), and TNSS (*I* ^2^ = 48.6%).

#### 3.4.7 Cross‐cultural validity

No multiple group factor analysis or differential item functioning was performed in cross‐cultural validity studies. Nevertheless, we present the measurement properties per country reported in validation studies in Table S8. Importantly, this table does not include any Italian studies because they do not report on the measurement properties of interest.^16^, ^17^ The properties are consistent throughout the other four countries in which CARATkids has been validated, except for reliability, where there is some heterogeneity.^10^, ^14^, ^15^, ^18^

### 3.5 Interpretability and Feasibility

Interpretability is summarized in Table S9. One study reported the percentage of missing items,^14^ which was low (0.3%). One study^14^ provided data on participants achieving minimum (floor score) and maximum (ceiling score) scores, with floor scores ranging from 8.2% to 15.6% and ceiling scores from 0.9% to 1.8%. Feasibility is described in Table S10. CARATkids includes 13 questions. The time for its completion was not assessed in any of the included studies. Its use for individual purposes is free and does not require any prior authorization for clinical use.

## 4 DISCUSSION

This study represents the first systematic review of measurement properties for a PROM in pediatric asthma and allergic rhinitis in accordance with the recommendations of the COSMIN guidelines.^19^, ^23^ We found CARATkids to display good content validity, internal consistency, reliability, construct validity, and responsiveness. These results suggest it is an appropriate instrument for use in the evaluation of asthma and allergic rhinitis control in children aged 6 to 12 years.

CARATkids was originally developed in Portuguese, using CARAT^13^ for adults as the base for its preliminary version. Its development was conducted by a panel of 26 specialists in asthma and allergic rhinitis, with diverse and complementary medical specialties.^11^ The version obtained in two consensus meetings was submitted to a cognitive test, leading to further modifications after feedback from children and their caregivers. However, the quality of its development was rated as “Inadequate,” following COSMIN guidelines, due to the lack of involvement of children and caregivers in PROM development prior to the cognitive interview.^31^, ^32^ Importantly, other PROMs^33^, ^34^, ^35^, ^36^ available for asthma in children have also shown inadequate PROM development. Additionally, among these PROMs, CARATkids was the only PROM undergoing a cognitive interview study, which is important for evaluating comprehensibility and comprehensiveness of a PROM.^21^ This cognitive interview was carried out in a representative sample of the population for which CARATkids was developed.^11^, ^28^ In addition, considering that the development process was based on an analogous version in clinical use for adults and its widespread clinical application,^13^ we believe that this rating does not interfere with its appropriateness for clinical use.

Content validity refers to the extent to which an instrument adequately represents the construct it is designed to measure.^19^, ^23^ It is widely regarded as the most important measurement property of an instrument. According to the development study^28^ and the independent assessment by two authors, content validity was considered “Sufficient.” However, the certainty of evidence was considered to be “Very low,” since there are no content validity studies available. Indeed, most asthma PROMs have not had their content validity rigorously assessed.^37^, ^38^

Regarding internal consistency, for it to be considered “Sufficient,” Cronbach alpha must be ≥0.7 and structural validity must be at least “Sufficient” with low evidence.^19^ We found a meta‐analytical Cronbach alpha of 0.82 with low heterogeneity, suggesting good internal consistency. None of the studies, however, assessed its structural validity (i.e., none of the published studies carried out a factor analysis, principal component analysis, Rasch analysis, or item response theory analysis). Therefore, internal consistency was rated “Indeterminate.”

CARATkids displayed high reliability, albeit with substantial heterogeneity. A few factors might be accountable for that. First, one of the studies^18^ lacked a clearly defined interval between visits, ranging from 2 to 49 weeks, while other studies evaluating reliability presented much shorter ranges: either 3 to 6 weeks^14^ or 4 to 6 weeks apart.^10^, ^15^ Additionally, the same study collected data in three different visits, while the other applied the questionnaire in two visits only.

There is no comparable gold standard assessing the control of asthma and allergic rhinitis. Therefore, we considered the comparisons between CARATkids and other validated PROMs to be evidence for construct validity (namely convergent validity), as construct validity reflects the degree to which the scores of a PROM are consistent with a priori hypotheses.^19^, ^23^ Except for VAS nose and EQ‐5D‐Y, we observed a strong correlation between CARATkids and the outcome measurement instruments to which it was compared, namely VAS global, VAS asthma, cACT, and the TNSS. Regarding EQ‐5D‐Y, a weaker correlation was expected, since this tool does not measure asthma or allergic rhinitis control, but rather health‐related quality of life, which is a related but dissimilar construct.^39^ Importantly, substantial heterogeneity was found in the comparisons with VAS asthma, cACT, and TNSS.

Our meta‐analysis pointed to good responsiveness when compared with VAS global, VAS nose, cACT, and TNSS, but important heterogeneity was observed for cACT. Given CARATkids strong internal consistency and validity properties, it is possible that its growing use could yield more evidence for responsiveness in future studies.

Regarding cross‐cultural validity, CARATkids has been translated, culturally adapted, and clinically validated for Brazilian Portuguese,^15^ Dutch,^18^ and Turkish,^10^ but no results on measurement invariance were reported, precluding the assessment of cross‐cultural validity. Nevertheless, we found consistent results reported in validation studies performed in different countries.

Regarding interpretability and feasibility, CARATkids was reported to be easy to understand by both patients and physicians, requiring only basic literacy and commonly available resources. It is also freely available for clinical use, supporting its applicability for assessing asthma and allergic rhinitis control in routine care.

Finally, we highlight that a recent study validated the electronic version of CARATKids against the gold standard paper‐based version, with the authors finding comparable internal consistency and reliability of the electronic version, in addition to a strong correlation between the electronic and paper versions, reflecting high construct validity.^30^ The validation of an electronic version together with this synthesis of measurement properties puts this PROM well positioned to be used in clinical studies.

This systematic review has some limitations worth noting. Firstly, the absence of a gold standard for the assessment of allergic rhinitis and asthma control precluded us from assessing criterion validity. We did, however, assess the construct validity (convergent validity) in comparison with other PROMs available for allergic rhinitis and asthma. Secondly, substantial heterogeneity was found for most analyses on construct validity and responsiveness. In many cases, however, heterogeneity decreased in leave‐one‐out sensitivity analysis without impacting the main conclusions. Third, the small number of included primary studies precluded us from following additional approaches for identification of sources of heterogeneity (such as meta‐regression). Besides that, it was not possible to assess structural validity, since none of the included studies evaluated this property. Finally, there is insufficient evidence on the quality of other PROMs used in allergic rhinitis or asthma, including on their development and content validity.^37^ Therefore, there is a need for further COSMIN‐based systematic reviews with meta‐analysis on other PROMs used in asthma and/or allergic rhinitis for pediatric patients.

This study presents important strengths. There have been previous systematic reviews of PROMs for allergic rhinitis or asthma in a pediatric population; however, they either did not assess the development quality of the PROMs, did not follow COSMIN recommendations,^4^, ^8^ or did not perform a meta‐analysis of measurement properties.^40^ The evidence obtained from this systematic review supports the use of CARATkids in clinical practice. Additionally, we performed a thorough literature search, including manually searching Google Scholar and screening references of included studies to minimize the risk of missing relevant publications. Finally, we were able to perform a meta‐analysis of most measurement properties.

In conclusion, this is the first systematic review with meta‐analysis to evaluate and summarize the measurement properties of a pediatric PROM designed for assessing the control of allergic rhinitis and asthma simultaneously. The findings indicate that CARATkids presents good content validity. Strong internal consistency and good reliability were also observed, albeit with low certainty of evidence. Responsiveness and construct validity also showed good results, with at least moderate evidence for most correlations. Overall, these findings support the suitability of CARATkids for assessment of asthma and allergic rhinitis in children from 6 to 12 years who present both diseases simultaneously. Future studies may aim to further validate the content and structural validity of CARATkids. Furthermore, additional systematic reviews of other PROMs used in the assessment of pediatric allergic rhinitis or asthma would support more comprehensive comparisons across available instruments.

## AUTHOR CONTRIBUTIONS

**Hadla Sami El Didi:** Data curation; formal analysis; investigation; visualization; writing – original draft; writing – review and editing. **Ana Margarida Pereira:** Writing – review and editing. **Cristina Jácome:** Writing – review and editing. **Rita Amaral:** Writing – review and editing. **Gustavo F. Wandalsen:** Writing – review and editing. **Joyce Emons:** Writing – review and editing. **Stefania La Grutta:** Writing – review and editing. **Giovanna Cilluffo:** Writing – review and editing. **Sehra Birgül Batmaz:** Writing – review and editing. **Daniela Linhares:** Writing – review and editing. **Dirceu Sole:** Writing – review and editing. **Bernardo Sousa‐Pinto:** Writing – original draft. **João Almeida Fonseca:** Supervision; writing – review and editing. **Rafael José Vieira:** Conceptualization; data curation; formal analysis; investigation; supervision; writing – original draft; writing – review and editing.

## CONFLICT OF INTEREST STATEMENT

Ana Margarida Pereira, Cristina Jácome, Rita Amaral, Gustavo F. Wandalsen, Joyce Emons, Stefania La Grutta, Giovanna Cilluffo, Sehra Birgül Batmaz, Daniela Linhares, Dirceu Sole, and João Almeida Fonseca were involved in the original studies of the development, validation, and/or mobile phone adaptation of CARATkids.

## PEER REVIEW

The peer review history for this article is available at https://www.webofscience.com/api/gateway/wos/peer‐review/10.1111/pai.70191.

## Supporting information


Data S1

Data S2
