# Supplementary material for: Control of Allergic Rhinitis and Asthma Test for Children (CARATkids): A systematic review and meta‐analysis of its measurement properties
Source: Pediatr Allergy Immunol. 2025 Sep 2;36(9):e70191. doi: 10.1111/pai.70191 (PMC12405605; doi:10.1111/pai.70191)
Supplement: Supplementary file 1 — Data S1 [file PAI-36-e70191-s002.docx]

**Supplementary Table 1. List of queries used for searching electronic databases.**

| **Number** | **Query** |  |
| --- | --- | --- |
| **Medline via OVID** | | |
| #1 | exp Asthma/ |  |
| #2 | (asthma$ or asthmat$).mp. |  |
| #3 | Bronchial Hyperreactivity/ |  |
| #4 | exp Bronchoconstriction/ |  |
| #5 | Bronchial Spasm/ |  |
| #6 | bronchospas$.mp. |  |
| #7 | (bronch$ adj3 spas$).mp |  |
| #8 | bronchoconstrict$.mp. |  |
| #9 | (bronch$ adj3 constrict$).mp. |  |
| #10 | ((bronchial$ or respiratory or airway$ or lung$) adj3 (hypersensitiv$ or hyperreactiv$ or allerg$ or insufficiency)).mp. |  |
| #11 | ((dust or mite$) adj3 (allerg$ or hypersensitiv$)).mp. |  |
| #12 | wheez$.mp. |  |
| #13 | 1 or 2 or 3 or 4 or 5 or 6 or 7 or 8 or 9 or 10 or 11 or 12 |  |
| #14 | exp Rhinitis/ |  |
| #15 | (rhinit$ or rhinoconjunctivit$).mp. |  |
| #16 | exp Allergic Rhinitis/ |  |
| #17 | (hayfever or "hay fever" or pollenosis or pollinosis or SAR).mp. |  |
| #18 | 14 or 15 or 16 or 17 |  |
| #19 | (CARAT or CARAT?10 or CARATkids).mp. |  |
| #20 | (control$ adj2 allerg$ adj2 rhinit$ adj2 asthm$ adj2 kids).mp. |  |
| #21 | 19 or 20 |  |
| #22 | (13 or 18) and 21 |  |
| **ISI Web of Science** | | |
| #1 | (TS=(asthma* OR asthmatic*) OR TS=(bronchospasm*) OR TS=(bronch* NEAR/3 spasm*) OR TS=(bronchoconstrict*) OR TS=(bronch* NEAR/3 constrict*) OR TS=((bronchial* or respiratory or airway* or lung*) NEAR/3 (hypersensitiv* or hyperreactiv* or allerg* or insufficiency)) OR TS=((dust or mite*) NEAR/3 (allerg* or hypersensitiv*)) OR TS=(wheez*)) |  |
| #2 | (TS=(rhinit* or rhinoconjunctivit*) OR TS=(hayfever or "hay fever" or pollenosis or pollinosis or SAR)) |  |
| #3 | (TS=(CARAT or CARAT$10 or CARATkids) OR TS=(control* NEAR/2 allerg* NEAR/2 rhinit* NEAR/2 asthm* NEAR/2 kids)) |  |
| #4 | (#1 OR #2) AND #3 |  |
| **Scopus** | | |
| #1 | ( TITLE-ABS-KEY(asthma* OR asthmatic*)  OR TITLE-ABS-KEY(bronchospas*)  OR TITLE-ABS-KEY(bronch* pre/3 spas*)  OR TITLE-ABS-KEY(bronchoconstrict*)  OR TITLE-ABS-KEY(bronch* pre/3 constrict*)  OR TITLE-ABS-KEY((bronchial* or respiratory or airway* or lung*) pre/3 (hypersensitiv* or hyperreactiv* or allerg* or insufficiency))  OR TITLE-ABS-KEY((dust or mite*) pre/3 (allerg* or hypersensitiv*))  OR TITLE-ABS-KEY(wheez*))  OR ( TITLE-ABS-KEY(rhinit* or rhinoconjunctivit*)  OR TITLE-ABS-KEY(hayfever or "hay fever" or pollenosis or pollinosis or SAR))  AND ( TITLE-ABS-KEY(CARAT or CARAT10 or CARAT-10 or CARATkids)  OR TITLE-ABS-KEY(control* pre/2 allerg* pre/2 rhinit* pre/2 asthm* pre/2 kids) ) |  |

**Supplementary Table 2. COSMIN criteria for good measurement properties.**

| **Measurement property** | **Rating** | **Criteria** |
| --- | --- | --- |
| **Internal consistency** | + | At least low evidence for sufficient structural validity AND Cronbach’s alpha(s) ≥ 0.70 for each unidimensional scale or subscale |
|  | ? | Criteria for “At least low evidence for sufficient structural validity” not met |
|  | – | At least low evidence for sufficient structural validity AND Cronbach’s alpha(s) < 0.70 for each unidimensional scale or subscale |
| **Reliability** | + | ICC or weighted Kappa ≥ 0.70 |
|  | ? | ICC or weighted Kappa not reported |
|  | – | ICC or weighted Kappa < 0.70 |
| **Hypotheses testing for construct validity** | + | The result is in accordance with the hypothesis |
|  | ? | No hypothesis defined (by the review team) |
|  | – | The result is not in accordance with the hypothesis |
| **Responsiveness** | + | The result is in accordance with the hypothesis OR AUC ≥ 0.70 |
|  | ? | No hypothesis defined (by the review team) |
|  | – | The result is not in accordance with the hypothesis OR AUC < 0.70 |

AUC = area under the curve, ICC = intraclass correlation coefficient, + = sufficient, - = insufficient, ? = indeterminate

**Supplementary Table 3.** **Quality of the development of the Control of Allergic Rhinitis and Asthma Test for Children (CARATkids).**

| **CARATkids design** | General design requirements | **Clear construct** | Very good |
| --- | --- | --- | --- |
|  |  | **Clear origin of construct** | Very good |
|  |  | **Clear target population** | Very good |
|  |  | **Clear context of use** | Very good |
|  |  | **Representativeness of sample** | Inadequate^†^ |
|  | Concept elicitation |  | NA |
|  | **Total CARATkids design** | | **Inadequate**^‡^ |
| **Cognitive interview study** | Representativeness of sample | | Very good |
|  | Comprehensibility | | Doubtful |
|  | Comprehensiveness | | NA |
|  | **Total cognitive interview study** | | **Doubtful**^‡^ |
| **Total CARATkids development** | | | **Inadequate**^‡^ |

^†^ CARATkids was developed based on consensus meetings with experts; children were not involved in the design.

^‡^ Based on the lowest rating.

**Supplementary Table 4.** **Quality of the development of Childhood Asthma Control**

**Test (cACT), Childhood Asthma Questionnaires (CAQ-B), and Test for Respiratory and Asthma Control in Kids (TRACK)**

|  |  | **cACT** | **CAQ-B** | **TRACK** |
| --- | --- | --- | --- | --- |
| **General design requirements** | Clear construct | Very good | Very good | Very good |
|  | Clear origin of construct | Very good | Very good | Very good |
|  | Clear target population | Very good | Very good | Very good |
|  | Clear context of use | Very good | Very good | Very good |
|  | Representativeness of sample | Very good | Doubtful | Very good |
| **Concept elicitation** | Data collection method | Very good | Very good | Very good |
|  | Interviewers’ skills | Doubtful | Very good | Doubtful |
|  | Use of an interview guide | Doubtful | Doubtful | Very good |
|  | Recording of interviews | Doubtful | Doubtful | Doubtful |
|  | Data analysis approach | Doubtful | Doubtful | Adequate |
|  | Data coding | Inadequate | Inadequate | Inadequate |
|  | Data collection saturation | Adequate | Adequate | Doubtful |
| **Cognitive interview** |  | NA | NA | NA |
| **Total development quality** | | Inadequate | Inadequate | Inadequate |

**Supplementary Table 5. Content validity of the Control of Allergic Rhinitis and Asthma Test for Children (CARATkids).**

|  | **Rating of reviewers** | **Overall rating** | **Certainty of evidence** | |
| --- | --- | --- | --- | --- |
| **Relevance** |  | **+** | Very low |  |
| Are the included items relevant for the construct of interest? | **+** |  |  |  |
| Are the included items relevant for the target population of interest? | **+** |  |  |  |
| Are the included items relevant for the context of use of interest? | **+** |  |  |  |
| Are the response options appropriate? | **+** |  |  |  |
| Is the recall period appropriate? | **+** |  |  |  |
| **Comprehensiveness** |  |  | Very low |  |
| Are all key concepts included? | **+** |  |  |  |
| **Comprehensibility** |  | **+** | Very low |  |
| Are the PROM items appropriately worded? | **+** |  |  |  |
| Do the response options match the question? | **+** |  |  |  |
| **Content validity** |  | **+** | **Very low** |  |

+ = sufficient

**Supplementary Table 6.** **Overall qualitative rating and certainty of evidence for measurement properties of the Control of Allergic Rhinitis and Asthma Test for Children (CARATkids).**

|  | Linhares 2014 | Resende 2015 | Emons 2016 | Amaral 2017 | Batmaz 2018 | Cillufo 2019 | Mata 2025 | Overall | CoE |
| --- | --- | --- | --- | --- | --- | --- | --- | --- | --- |
| Internal consistency | ? |  | ? | ? | ? |  | ? | ? | Low |
| Reliability | + |  | + | + | + |  |  | + | Low |
| Measurement error |  |  | + | + | + |  |  | + | High |
| Construct validity: VAS nose | - |  | - | + | - |  |  | - | High |
| Construct validity: VAS asthma | + |  | + | + | + |  |  | + | Moderate |
| Construct validity: VAS global | |  | + |  |  |  |  | + | Moderate |
| Construct validity: TNSS |  |  |  | + | + |  |  | + | Low |
| Construct validity: cACT | + |  | + | + | + | + |  | + | Moderate |
| Construct validity: VAS EQ-5D-Y | | + |  |  |  |  |  | + | Low |
| Responsiveness: VAS nose | - |  | + | + | + |  |  | + | High |
| Responsiveness: VAS asthma | - |  | + | - | + |  |  | ± | Moderate |
| Responsiveness: VAS global |  |  | + |  |  |  |  | + | Moderate |
| Responsiveness: TNSS |  |  |  | - | + |  |  | ± | Moderate |
| Responsiveness: cACT | - |  | + | + | + | + |  | + | Moderate |

+ = sufficient; – = insufficient; ± = inconsistent; ? = indeterminate; CoE = Certainty of Evidence.

Tosca 2020 assessed construct validity by comparing CARATkids with cACT scores; however, the results were reported in a manner that did not allow for a COSMIN-compliant rating to be determined.

**Supplementary Table 7. Areas under the ROC curve of the Control of Allergic Rhinitis and Asthma Test for Children (CARATKids).**

|  | **cACT** | **VAS Asthma** | **VAS Rhinitis** | **TNSS** | **GINA** |
| --- | --- | --- | --- | --- | --- |
| Amaral 2017 | 0.97 (95% CI = 0.93 - 1.00) | 0.89 (95% CI = 0.79 - 0.99) | 0.76 (95% CI = 0.66 - 0.86) | 0.88 (95% CI = 0.82 - 0.95) | - |
| Batmaz 2018 | 0.89 (p<0.001) | 0.88 (p<0.001) | 0.80 (p<0.001) | 0.78 (p< 0.001) | 0.97 (p<0.001) |
| Cilluffo 2019 | 0.91 (95% CI = 0.82 - 1.00) | - | - | - | - |
| Linhares 2014 | 0.83 | 0.81 | 0.76 | - | - |

**Supplementary Table 8. Measurement properties reported in cross-cultural validation studies of the Control of Allergic Rhinitis and Asthma Test for Children (CARATkids).**

|  | **Internal consistency^†^** | **Reliability^‡^** | **Construct validity** **^§^** | | | **Responsiveness** **^§^** | | |
| --- | --- | --- | --- | --- | --- | --- | --- | --- |
|  |  |  | **VAS Nose** | **VAS Asthma** | **cACT** | **VAS Nose** | **VAS Asthma** | **cACT** |
| Portugal | 0.80 | 0.80 | 0.45 | 0.61 | -0.69 | 0.45 | 0.47 | -0.34 |
| Brazil | 0.81 | 0.85 | 0.58 | 0.51 | -0.76 | 0.51 | 0.30 | -0.57 |
| Netherlands | 0.80 | 0.61 | 0.49 | 0.56 | -0.57 | 0.52 | 0.57 | -0.51 |
| Turkey | 0.84 | 0.97 | 0.42 | 0.69 | -0.77 | 0.57 | 0.58 | -0.79 |

^†^ Cronbach's alpha. ^‡^ Intraclass correlation coefficient. ^§^ Spearman correlation coefficient. VAS = Visual Analogue Scale. cACT = Childhood Asthma Control Test.

**Supplementary Table 9. Interpretability of the Control of Allergic Rhinitis and Asthma Test for Children (CARATkids).**

|  |  | **Percentage of missing items or of missing total scores** | **Floor scores (%)^†^** | **Ceiling scores (%)^‡^** | **Subgroups data** | **MIC/MID** | **Completion time** |
| --- | --- | --- | --- | --- | --- | --- | --- |
| Linhares 2014 |  | Information for the 17 item version. Percentage of missing items 0.3% (overall). The items with more missing items each with two missings (1.8%) were ‘Wheezing’, by both children and parents, ‘Rhinorrhoea’ and ‘Throat symptoms’ by children, and ‘Nasal Obstruction’ and ‘Dyspnoea’ by parents | 8.2 (visit 1)  15.6 (visit 2) | 1.8 (visit 1)  0.9 (visit 2) | Gender; GINA and ARIA classifications and by physician treatment decision. Asthma and rhinitis severity as per physician classification, GINA classification of asthma control, ARIA classification of severity and control groups defined by cACT; Treatment decision groups | – | – |
| Emons 2017 |  | Response rate was 86% for the second visit and 79% for the third. However, this includes both children and adolescents subgroups. There was no report regarding missing items. Drop in response rate was attributed to discharge, loss of follow up and, for a small percentage of patients, to incompleteness of the questionnaire. | – | – | Age (all patients, <12 years and ≥12 years); Asthma control (according to ACT); Rhinitis control (according to VAS) | 2.76 | – |
| Amaral 2017 |  | – | – | – | Asthma control (defined by cACT, GINA and VAS Asthma); Rhinitis severity (defined by TNSS and VAS-rhinitis) | 3 | – |
| Batmaz 2018 |  | – | – | – | Asthma control (according to GINA classification, cACT score, VAS asthma), Rhinitis severity (according to ARIA classification, VAS rhinitis and TNSS) | 4 | – |
| Cilluffo 2019 |  | – | – | – | – | – | – |
| Tosca 2020 |  | – | – | – | Age (Children and adolescents); GINA classification of asthma control; cACT assessment of asthma control; CARATkids assessment of asthma and acute rhinitis control | – | – |
| Mata 2025 |  | – | – | – | Children and caregiver | – | – |

^†^ Percentage of users obtaining the minimum score in the questionnaire. ^‡^ Percentage of users obtaining the maximum score in the questionnaire. ARIA = Allergic Rhinitis and Its Impact on Asthma (ARIA); cACT = Childhood Asthma Control Test; GINA = Global Initiative for Asthma MIC = Minimal important change; MID = Minimal important difference; TNSS = Total Nasal Symptom Score; VAS = Visual Analogue Scale.

**Supplementary Table 10. Feasibility of the Control of Allergic Rhinitis and Asthma Test for Children (CARATkids) questionnaire.**

| Patients' comprehensibility | Children (6-12 years old) |
| --- | --- |
| Clinicians' comprehensibility | Any clinician |
| Type and ease of administration | Paper or online (mobile) |
| Length of the instrument | 13 questions |
| Completion time | – |
| Patients' required mental and physical ability level | No required specific physical ability.  Basic literacy |
| Ease of score calculation | Easy (sum of points for each question) |
| Copyright | This questionnaire is copyrighted mostly to prevent unapproved changes to it. Its use for individual purposes (e.g. supporting clinical assessment at a patient consultation) is free and does not requires any authorization. The use of the questionnaire by any research group, to aggregate data from different patients, requires a communication to the CARATkids group. For-profit organizations or the use of the questionnaire with commercial/marketing purpose require case-by-case authorization from the CARATkids group. |
| Cost of an instrument | None for clinical care and academic studies (may have costs for other uses) |
| Required equipment | None other than paper and pen OR smartphone |
| Availability in different settings | Available in any setting (clinical setting or home) |
| Regulatory agency's requirement for approval | Not required |

**Supplementary Figure 1. COSMIN Flowchart for conducting a systematic review of patient-reported outcome measures (PROMs).**

**B. Evaluate the measurement properties**

**C. Select a PROM**

**A. Perform the literature search**

1. **Formulate the aim of the review**
2. **Formulate eligibility criteria**
3. **Perform a literature search**
4. **Select abstracts and full-text articles**
5. **Evaluate content validity**
6. **Evaluate internal structure**

- Structural validity
- Internal consistency
- Cross-cultural validity

1. **Evaluate the remaining measurement properties**

- Reliability
- Measurement error
- Criterion validity
- Hypotheses testing for construct validity
- Responsiveness

1. **Evaluate interpretability and feasibility**
2. **Formulate recommendations**
3. **Report the systematic review**

**Evaluate the quality of the PROM:**

- Evaluate the methodological quality of the included studies by using the **COSMIN Risk of Bias checklist**
- Apply criteria for good measurement properties by using **quality criteria**
- Summarize the evidence and grade the quality of the evidence by using the **GRADE approach**
